# Effects of T-2 toxin on digestive enzyme activity, intestinal histopathology and growth in shrimp *Litopenaeus vannamei*

**DOI:** 10.1038/s41598-019-49004-4

**Published:** 2019-09-11

**Authors:** Zhanrui Huang, Yaling Wang, Mei Qiu, Lijun Sun, Yijia Deng, Xiaobo Wang, Siyuan Bi, Ravi Gooneratne, Jian Zhao

**Affiliations:** 1 College of Food Science and Technology, Guangdong Ocean University, Guangdong Provincial Key Laboratory of Aquatic Product Processing and Safety, Key Laboratory of Advanced Processing of Aquatic Products of Guangdong Higher Education Institution, Zhanjiang, 524088 China; 2 Ski Teaching and Training Base Post-doctoral Research Station of Harbin Sport University, Harbin, 150008 China; 3 Department of Wine, Food and Molecular Biosciences, Faculty of Agriculture and Life Sciences, Lincoln University, Lincoln, 7647 Canterbury New Zealand; 4 School of Chemical Engineering, The University of New South Wales, Sydney, NSW 2052 Australia

**Keywords:** Enzyme mechanisms, Toxicology, Diseases

## Abstract

T-2 toxin (T-2), a naturally occurring mycotoxin that often accumulates in aquatic animals via contaminated feed, is toxic to animals, including humans. In this study, six groups of shrimp (n = 30 shrimps/group) were given T-2 in feed at concentrations of 0–12.2 mg/kg for 20 days. T-2 accumulation, intestinal histopathology, digestive enzyme activities and subsequent effects on shrimp are reported. Compared to the control, T-2 significantly reduced weight gain, specific growth rate, and survival. The histopathology of shrimp intestine showed concentration-dependent degenerative and necrotic changes in response to dietary T-2. Progressive damage to the microstructures of shrimp intestine occurred with increasing dietary T-2 concentrations, with initial inflammation of the mucosal tissue at T-2 concentrations of 0.5 and 1.2 mg/kg, progressing to disappearance of intestinal villi and degeneration and necrosis of the submucosa at 12.2 mg/kg. Intestinal amylase and protease activities increased at low T-2 concentrations but showed significant inhibition at high concentrations; however, the opposite trend occurred for lipase activity. Collectively, these results indicate that digestive enzyme activities and mucosal structures are markedly affected by exposure to T-2, and these may have contributed to the lower survival rate of shrimp.

## Introduction

With the rapid development of aquaculture, *Litopenaeus vannamei* has become one of the largest shrimp exports from China to the world^1,2^. In the past decade, the production of shrimp in aquaculture has intensified and the industry expanded extensively. At the same time, mycotoxin contamination of aquatic feed has increased because more cereal types with high protein are now incorporated into these feeds, replacing animal proteins to reduce feed costs^3,4^.

T-2 toxin (T-2) is among the most toxic of the trichothecene mycotoxins, a large group of compounds produced by several *Fusarium* species that occur in mold-damaged foods around the world^5,6^. T-2 is rapidly absorbed by aquatic animals and causes a wide range of toxic effects^7^. Ingestion of T-2 by aquatic organisms has been found to damage the stomach, hepatopancreas and intestinal mucosa, and reduce feed intake and growth^8,9^.

As a food safety measure, the residues of T-2 in food and feeds are closely monitored^10^. Extensive research has explored the mechanisms of T-2 toxicity in humans and animals, with inhibition of protein synthesis, damage to digestive tract and reduction in immunity being the main mechanisms found. For example, in ducks fed T-2 containing feed for three weeks, the rate of weight gain was significantly reduced, and the digestive tract was severely damaged^11^. On exposure of catfish to T-2 at 1.0 mg/kg in the diet, intestinal immunity declined, and mortality increased up to 84%^12^. In *Litopenaeus vannamei* and *Penaeus monodon* given a diet containing T-2 at 1.0–2.0 mg/kg for up to 10 weeks, the digestive tract mucosa was severely inflamed^13^.

Shrimp intestine is not only a digestive organ, but also an important part of the immune system^14^. Several animal studies have evaluated the effects of a range of nutrients on intestinal structure^15–17^. However, relatively few studies have explored the influence of mycotoxins (especially T-2) on intestinal histopathology. Furthermore, it was found that the effects of T-2 on shrimp intestinal histopathology have not been reported. Intestinal digestive enzyme activities during shrimp growth have been studied^18–20^. Protease, amylase and lipase play a key role in food digestion and nutrient absorption from the intestine^21,22^. Effects of T-2 on shrimp digestive enzymes have not been reported.

In our previous research, we have found that T-2 damaged the microstructure of shrimp hepatopancreas in a concentration-dependent manner and had a significant effect on alkaline phosphatase (AKP), glutamic-oxaloacetic transaminase (GOT) and glutamic-pyruvic transaminase (GPT) activities^23^. And the effects of T-2 on the survival rate of shrimp weighing 3.5 ± 0.5 g and 8.5 ± 0.5 g were significantly different^23,24^. In this study, shrimp with a body weight of 5.0 ± 0.5 g were studied. Growth parameters, intestinal histopathology and digestive enzyme activities were analyzed to better understand the toxic effects of T-2 in shrimp.

## Results

### Growth parameters of shrimp exposed to T-2 toxin

**Figure 1:**
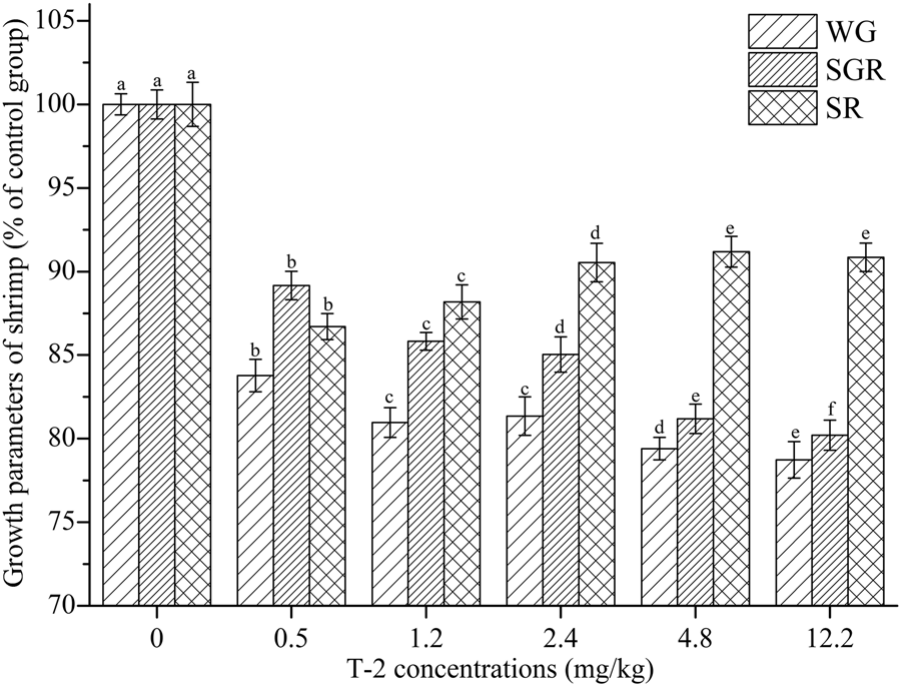
Reductions in growth parameters and survival as a function of dietary concentrations of T-2 toxin. Different superscripts indicate significant differences. Weight gain is shown as WG, specific growth rate is shown as SGR, and survival rate is shown as SR.

Growth parameters of shrimp (n = 30/group) exposed to T-2 are presented in Fig. 1. Compared to the control group, all growth parameters of T-2 dosed shrimp declined significantly. As the concentration of T-2 increased, the weight gain rate and specific growth rate of shrimp gradually decreased. The survival rate showed a highly significant difference between treatments and control (*P* < 0.05). The worst survival rate was observed in the group given 1.2 mg/kg. However, the survival rate began to rise gradually with higher T-2 concentrations in the feed.

### Effects of T-2 toxin on shrimp intestinal histopathology

**Figure 2:**
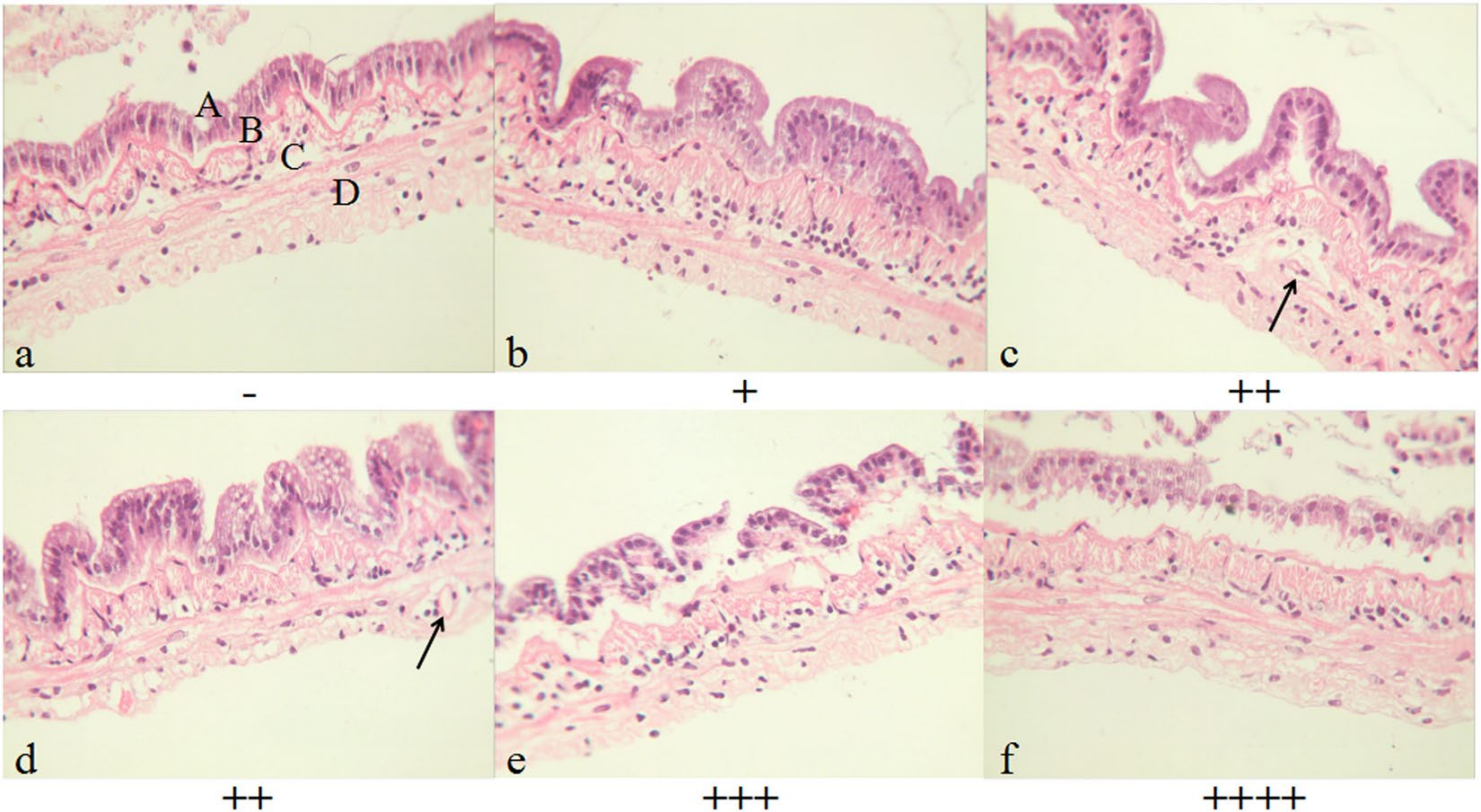
Histopathology of shrimp intestine exposed T-2 (×400). Description of letter abbreviations: A- Striated border, B- Mucosa, C- Submucosa, and D- Muscular layers. Figures **a** to **f** are the shrimp given 0 (control), 0.5, 1.2, 2.4, 4.8, 12.2 T-2 mg/kg respectively.

The criteria for evaluation of the extent of damage caused by T-2 toxin on shrimp intestinal histopathology are shown in Table 1. According to the criteria, the degree of damage was divided into 5 levels: normal (−), minimal (+), mild (++), moderate (+++) and severe (++++). The microstructures of shrimp intestine in the control and test groups (n = 5) are shown in Fig. 2. The shrimp intestine in the control group exhibited a well-defined striated border, complete mucosal folds and a clear organelle structure (Fig. 2-a). In the 0.5 and 1.2 mg/kg T-2 concentration groups, the shrimp intestine appeared inflamed with enlarged striated edge and shorter mucosal folds (Fig. 2-b,c). In the 2.4 mg/kg group, the intestinal submucosal space was increased, the villi were almost non-existent, and the mucosal folds were much shorter (Fig. 2-d). In the 4.8 mg/kg group, the intestine was swollen, the striated border was missing, and the intercellular space was even larger, so that the layers were separated (Fig. 2-e). In the highest T-2 concentration group (12.2 mg/kg), the intestine was severely damaged, the intestinal villi had disappeared, mucosal folds were extremely short, and the submucosa had undergone marked degeneration and necrosis (Fig. 2-f).

**Table 1:** Criteria used to evaluate the degree of damage to shrimp intestine.

Damage degree	Criteria	Intestinal structure				
		A	B	C	D	E
		Striated border	Mucosa	Submucosa	Muscular layer	Whole structure
−	Normal	Arranged closely	Folds, villi & crypts normal	Tight intercellular space	Cells arranged in order	Clear and complete
+	Minimal	Arranged closely	Shorter folds & short villi	Large spaces	Space enlarged	Loose structure
++	Mild	Swelling	Less folds, Shorter villi	Large spaces	Vacuolation & dispersed	Loose tissue
+++	Moderate	Degeneration	Folds almost disappeared, Very short villi	Not compact or dense	Marked vacuolation, Nucleolysis	Necrosis
++++	Severe	Exfoliation	Disintegration of villi, some necrosis	Dissolution	Separation	Necrosis

A, B, C, D refers to different parts of the intestine as referred to in Fig. 2.

### Effects of T-2 toxin on shrimp digestive enzyme activities

**Figure 3:**
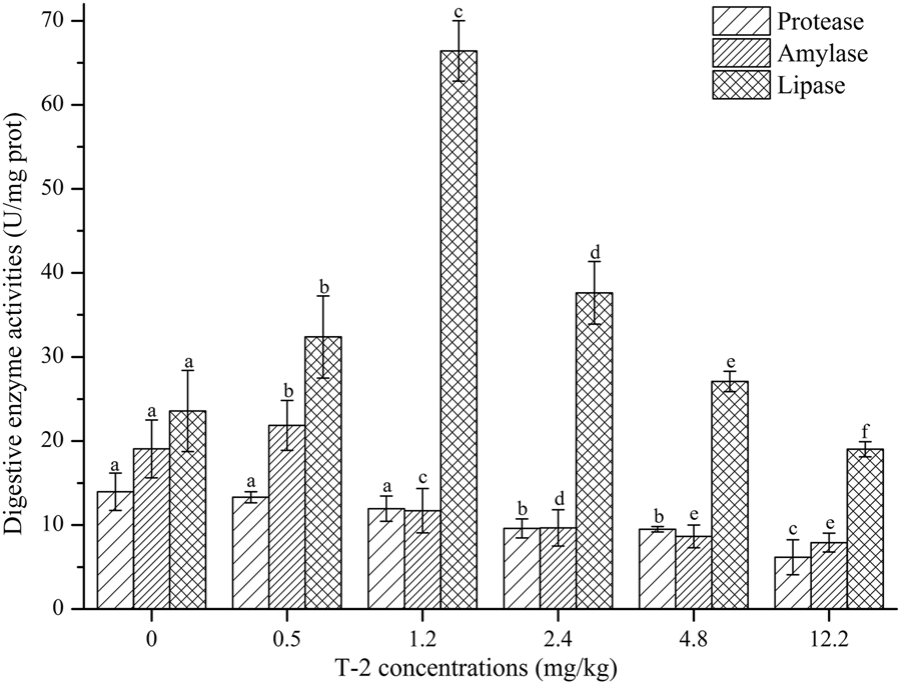
The effect of T-2 on digestive enzyme activities (Mean + SD) in shrimp intestine. Bars with different superscript letters for each enzyme are significantly different (*P* < 0.05) compared with the control (0).

The effects of different T-2 concentrations on shrimp intestinal digestive enzymes are shown in Fig. 3. With increasing concentrations of T-2, the activity of intestinal protease and amylase decreased and this would have reduced protein and carbohydrate digestion. The reason for the reduction in protease activity may be that T-2 inhibited the expression of protein. Significant differences (*P* < 0.5) were observed in intestinal lipase activity. It was highest with T-2 at 1.2 mg/kg of feed. As T-2 concentrations increased to higher levels, step-wise reductions in lipase activity were noted, but only the highest (12.2 mg/kg) concentration was associated with activity that was significantly below that of the control.

### Concentration-response relationships between T-2 toxin and digestive enzyme activities

**Figure 4:**
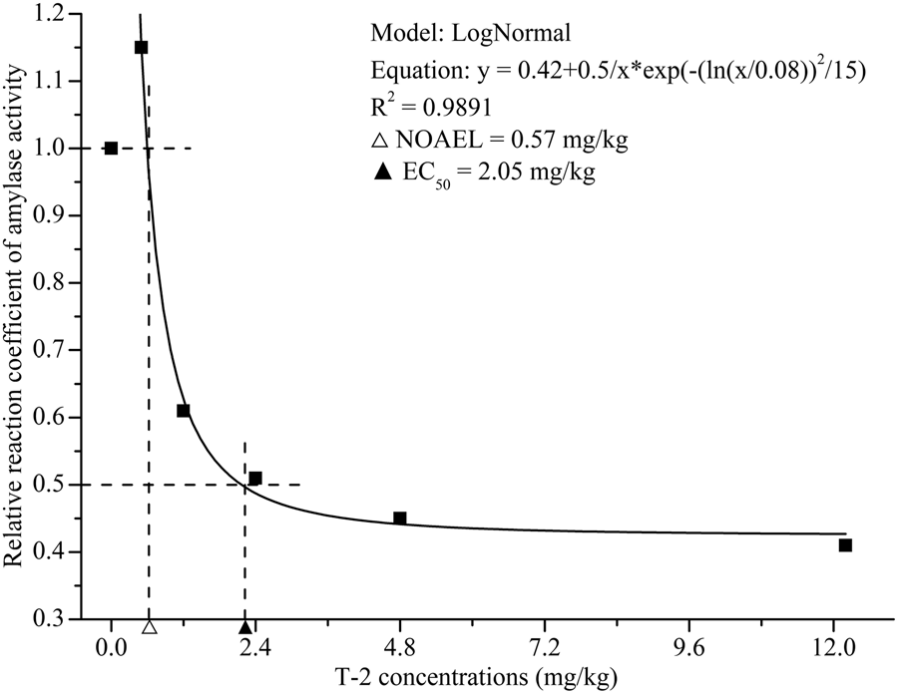
Effect of T-2 exposure concentration on the ratio of amylase enzyme activity (treatment group/control group) in shrimp intestine. NOAEL: No Observable Adverse Effect Level; EC_50_: T-2 concentration for 50% of amylase enzyme activity.

**Figure 5:**
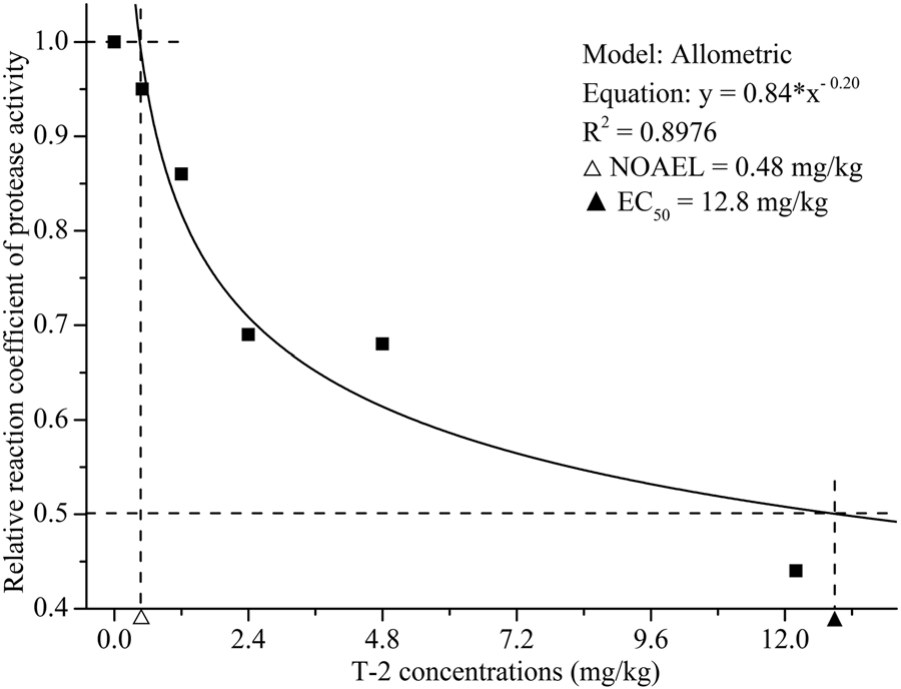
Effect of T-2 exposure concentration on the ratio of intestinal protease enzyme activity (treatment group/control group) in shrimp intestine. NOAEL: No Observable Adverse Effect Level; EC_50_: T-2 concentration for 50% of protease enzyme activity.

**Figure 6:**
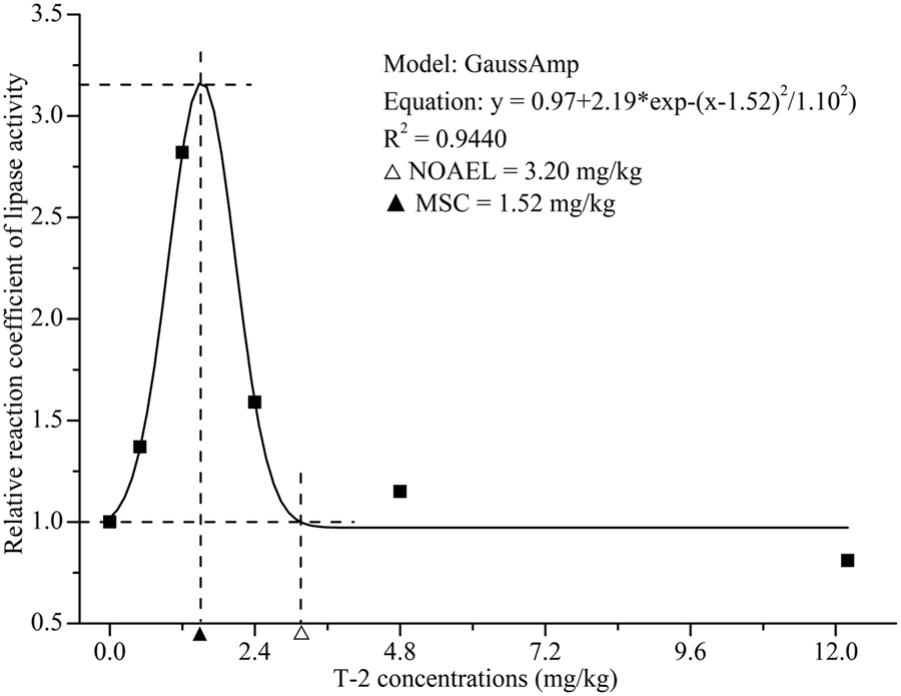
Effect of T-2 exposure concentration on the ratio of intestinal lipase enzyme activity (treatment group/control group) in shrimp intestine. NOAEL: No Observable Adverse Effect Level; MEC: T-2 concentration of maximal lipase enzyme activity.

Concentration-response correlations illustrate the relationships between intestinal enzyme activities expressed as the ratio between the test and control groups in the y-axis and T-2 concentrations in feed (mg/kg) in the x-axis (Figs 4–6). The activities of intestinal protease, amylase and lipase were consistent when analyzed using Allometric^25^, LogNormal^26^ and GaussAmp models^27^, respectively. The concentration-response correlations between the T-2 concentration and digestive enzyme activities were high (*R*^2^ = 0.8976 to 0.9891), and variance analysis showed that the differences were significant. Intestinal amylase exhibited the minimal EC_50_ induced by T-2. In contrast to protease and amylase, at concentrations between 0.5 and 3.2 mg/kg, T-2 had a stimulating effect on lipase activity, and the highest lipase activity occurred at the T-2 concentration of 1.52 mg/kg.

## Discussion

Contamination of feed ingredients with toxic concentrations of mycotoxins has been so serious in recent years, that it seems likely to limit the expansion of aquaculture in Asia^28,29^. It has been shown that dietary aflatoxin B_1_ caused poor growth performance and deformities of juvenile grass carp^30^. Dietary mixtures of aflatoxin B1 and fumonisin B1 resulted in reduction in growth performance in juvenile catfish^31^. Weight gain and survival of shrimp were impaired by dietary deoxynivalenol after five weeks of exposure^32^. It is clear that mycotoxins can inhibit the growth of shrimp. In this study, shrimp (5.0 ± 0.5 g) were exposed to different T-2 concentrations in feed for 20 days, and it had a major impact on the weight gain rate and specific growth rate of shrimp after 20 d of exposure. Both of these parameters declined in shrimp given T-2 at any concentration, responses that reflect toxicity. However, it was very interesting that the survival rate of shrimp was not consistent with the theoretical speculation. The group exposed to the lowest T-2 concentration was associated with the most severe impact on survival. This might have been due to the higher concentrations of T-2 inducing damage to the intestine that was severe enough to reduce further T-2 absorption and thus systemic toxicity. By contrast, in our previous research with shrimp weighing 8.5 + 0.5 g, there was a modest decline in survival rate with T-2 in feed at concentrations of 1.2 mg/kg and greater^24^. In our various studies with dietary T-2, survival was the lowest (72%) in shrimp that weighed 3.5 + 0.5 g that were given the toxin at 1.2 mg/kg of feed^23^. In addition, Bundit *et al*.^33^ also discovered that T-2 inhibited black tiger shrimp (average weight = 4.7 g) survival rate even at 0.1 mg/kg. Combined with the results of this study, it seems that low T-2 concentrations have a marked inhibitory effect on the survival rate of juvenile shrimp (average weight ~ 5.0 g).

Shrimp intestine is a digestive organ and an important part of the immune system that plays a major role in nutrient and also toxin absorption^14,34^. Because the gastrointestinal mucosa is the first barrier between the body and orally ingested exogenous compounds, it has developed various mechanisms to limit absorption of toxins^35^. Studies have shown that T-2 not only reduces the shrimp growth but also can cause marked changes to the digestive system structure^9^. Supamattaya *et al*.^13^ have shown that feeding *Litopenaeus vannamei* and *Penaeus monodon* a diet containing T-2 at 1.0–2.0 mg/kg for 8 and 10 weeks can cause serious degeneration and atrophic changes in the intestines. T-2 can induce gross and histologic changes in the intestine of rats^36^. T-2 altered intestinal morphology in turkeys with resultant shorter and thinner villi^37^. In our study, marked intestinal tissue damage was evident as T-2 concentration increased. In the high T-2 concentration group, the shrimp intestinal tract was severely damaged, where almost all of the intestinal villi were detached or non-existent, the mucosal structure was loose, and the submucosa had partially undergone dissolution. Such drastic damage will affect shrimp health by reducing nutrient digestion and absorption. It is inferred that there was a direct relationship between the damage to the intestinal structure and shrimp survival rate.

There were changes in the activities of three key enzymes, protease, lipase and amylase, which could alter shrimp nutrient metabolism^38,39^. Studies have shown that low concentrations of certain mycotoxins in feed can stimulate the activity of digestive enzymes in experimental animals. For example, aflatoxin B_1_ at 2.5 mg/kg in the diet of chickens increased amylase activity, and 40 μg/kg increased both amylase and protease activity^40,41^. Digestive enzymes of broiler chickens were also influenced by T-2^42^. These results are similar to the trend of intestinal digestive enzymes of shrimp observed in the low T-2 concentration groups of this study. The activities of the three digestive enzymes increased slightly at T-2 concentrations of 0.5 and 1.2 mg/kg with lipase reaching a maximum of 66.42 ± 3.60 U/mg protein in the shrimp fed a diet containing T-2 at 1.2 mg/kg. We postulate that shrimp were stressed by the two lower concentrations of T-2 in a manner that increased lipase secretion. However, protease and amylase activities were decreased in response to higher concentrations of T-2, and those changes might be attributable to reduced protein synthesis.

Based on the concentration-response relationship between T-2 and digestive enzyme activities, the correlation coefficients of the concentration-response curves were high (R^2^ = 0.8976 to 0.9891), indicating that the concentration-response curves fitted the experimental data well. The NOAEL values of intestinal protease and amylase activities were ~0.50 mg/kg, indicating that T-2 can inhibit protease and amylase at fairly low concentrations^43^. Comparing the curve equation of the digestive enzymes, the EC_50_ for T-2 toxin-induced intestinal amylase activity was the lowest. This means that as a biomarker of T-2 intoxication, amylase would be more sensitive than intestinal protease and lipase of shrimp.

Rotter *et al*.^44^ and Awad *et al*.^45^ found that deoxynivalenol, a type B trichothecene, can cause necrosis of the digestive tract mucosa, which would then seriously impact on the healthy growth of animals. When digestive enzymes are induced by exogenous compounds and thus stimulate the activity of the host’s natural digestive enzyms, it can lead to a change in shrimp growth^38,46^. Some mycotoxins can decrease digestive enzyme activity as well as other digestive functions in animals, and thereby inhibit their growth and development^47,48^. The survival rate of shrimp sharply decreased in the T-2 high concentration groups. This appears to suggest that intestinal tract inflammation, intestinal wall thinning, shrinkage of intestinal villi and folds, and inhibition of key digestive enzymes collectively caused significant changes in shrimp digestive function, resulting in reduced survival of the organism.

## Methods

### Animals and chemicals

*Litopenaeus vannamei* (5.0 ± 0.5 g) were purchased from East Island (Zhanjiang, China). T-2 (purity ≥98%) was purchased from Enzo (USA). All other chemicals (Analytical reagents) used in the study were obtained from Qiyun Biological Technology (Guangzhou, China).

### Shrimp toxicity study

T-2 mixed shrimp feed was prepared according to Dai *et al*.^49^. Shrimp were divided into six groups (30 shrimps/group) and placed in six water tanks (75 × 60 × 50 cm; water volume: 150 L) for 7 d for them to adapt to the conditions (pH: 7.5 ± 0.1; water temperature: 25 ± 1 °C; salinity: 10‰; dissolved oxygen: 7.0–7.5 mg/L). According to the increasing concentration grouping paradigm of 20 d accumulation toxicity test, the concentrations of T-2 used were 0 (control), and 1/50, 1/20, 1/10, 1/5 and 1/2 LC_50_ (LC_50_ = 24.4 mg T-2/kg feed)^50^, thus the concentrations were 0 (control), 0.5, 1.2, 2.4, 4.8 and 12.2 mg T-2/kg feed respectively. Shrimp were fed three times a day (total daily feed intake ~ 5% of body weight) for 20 days^10,24^. One-third of the water in the tank was replaced with fresh water every morning. On day 21, the total weight of all shrimp in each tank was determined. Shrimp were anesthetized with ice, killed, and the midgut was removed and stored at −70 °C until required.

### Growth parameters of shrimp

The weight gain rate, survival rate and specific growth rate of shrimp (n = 30/group) in different treatment groups were calculated^51,52^ as follows:

$${\rm{Weight}}\,{\rm{gain}}\,{\rm{rate}}\,( \% )=({{\rm{W}}}_{{\rm{f}}}-{{\rm{W}}}_{{\rm{i}}})/{{\rm{W}}}_{{\rm{i}}}\times \mathrm{100};$$

$${\rm{Survival}}\,{\rm{rate}}\,( \% )=({{\rm{N}}}_{{\rm{i}}}-{{\rm{N}}}_{{\rm{f}}})/{{\rm{N}}}_{{\rm{i}}}\times \mathrm{100};$$

$${\rm{Specific}}\,{\rm{growth}}\,{\rm{rate}}\,( \% )=[({{\rm{lnW}}}_{{\rm{f}}}-{{\rm{lnW}}}_{{\rm{i}}})/{\rm{days}}]\times \mathrm{100};$$

where W_f_ and W_i_ are the final and initial average body weights on day 21 and 1 respectively. N_f_ and N_i_ are the final and initial (=50) number of shrimp in each group.

### Histopathology of intestine

The midgut of fresh shrimp intestine (n = 5) from each group was fixed in 10% neutral-buffered formalin for 24 h and dehydrated with a gradient of alcohol (50% to 95%). Next, intestines were embedded, sectioned using a microtome and stained as described by Qiu *et al*.^23^. Histopathologic changes in the intestine were observed using a light microscope (Olympus CKX41, Tokyo, Japan).

### Digestive enzyme analyses

Midguts (n = 5) of shrimp intestine from each group were homogenized (IKAT 25, Staufen, Germany) for 1 min (5000 × g) in cold distilled water and centrifuged (Himac CS150GXII, Hitachi, Tokyo) for 20 min (8,000 × g) at 4 °C. The supernatant was used to measure the digestive enzyme activities. Protease activity was determined by the casein-hydrolysis method of Furne *et al*.^53^. Amylase activity was determined by the starch-hydrolysis method of Zokaeifar *et al*.^54^. Lipase activity was determined according to the method of Muralisankar *et al*.^19^ by degrading triacylglycerol to free fatty acids. Digestive enzyme activities are expressed as U/mg of protein.

The concentration-response curves between T-2 and digestive enzyme activities in shrimp intestine were constructed using Origin 8.5. The curves were drawn with T-2 concentration (mg/kg) as the x-axis, and the ratio between the value of the experimental group and the control group (relative coefficient) as the y-axis. The NOAEL (no observable adverse effect level, concentration of T-2 when the ratio of enzyme activity was 1), MEC (maximal effect concentration of T-2, concentration of T-2 when the ratio of enzyme activity was maximum) and EC_50_ (concentration for 50% of maximal effect, concentration of T-2 when the ratio of enzyme activity was 0.5) were calculated by GraphPad Prism 7 (GraphPad Software, La Jolla, CA).

### Statistical analyses

Data are presented as the mean ± standard deviation (SD). All statistical analyses were conducted using GraphPad Prism 7, SPSS 22.0 (IBM, Chicago, USA) and Origin 8.5 (OriginLab, Massachusetts, USA). One-way ANOVA was performed and Duncan’ s multiple range test at a significant level of 0.05 was used to determine differences among groups.
